# Posterior fossa ependymoblastoma diagnosed in the second month of life: uneventful 12 years survival after gross total resection followed by chemotherapy

**DOI:** 10.1186/s40064-015-1178-1

**Published:** 2015-08-02

**Authors:** Bernt Johan Due-Tønnessen, Arild Egge, Tryggve Lundar, Bård Krossnes, Einar Stensvold, Paulina Due-Tønnessen, Petter Brandal

**Affiliations:** Department of Neurosurgery, Oslo University Hospital, Postboks 4950, Nydalen, 0424 Oslo, Norway; Department of Pathology, Oslo University Hospital, Oslo, Norway; Department of Pediatrics, Oslo University Hospital, Oslo, Norway; Department of Radiology, Oslo University Hospital, Oslo, Norway; Department of Oncology, Oslo University Hospital, Oslo, Norway

**Keywords:** Posterior fossa ependymoblastoma, Long-term survival, Pediatric neurosurgery

## Abstract

We report on an infant who underwent gross total resection (GTR) of a posterior fossa ependymoblastoma in the second month of life followed by chemotherapy with uneventful long-term survival for 12 years. Postoperative radiotherapy has been considered obligate to have a chance for prolonged survival, but is inadvisable in infants. To our knowledge, this is the first reported long-term survival in an infant treated for ependymoblastoma.

## Introduction

Ependymoblastoma is an exceedingly rare and highly malignant tumor of the CNS, occurring most frequently in infancy and early childhood (Ding et al. 2014). In the 2007 WHO classification of tumors of the CNS, ependymoblastoma is classified as an embryonal tumor and is primarily considered a sub-type of primitive neuroectodermal tumors (CNS PNETs, WHO grade IV). The majority of reported cases occur in supratentorial locations. Although it is a highly malignant tumor, there is currently no standardized treatment strategy for ependymoblastoma.

Clinical results remain very poor, even after multimodal treatment with surgical resection, chemotherapy and radiatiotherapy (Ding et al. 2014; Gerber et al. 2011; Mørk and Rubinstein 1985). We report a 2-month-old boy diagnosed with posterior fossa ependymoblastoma and his uneventful survival for 12 years following gross total resection (GTR) and adjuvant chemotherapy.

## Case report

This 2-month-old boy presented with a tense fontanelle, abnormal head growth, episodes of vomiting, and “sunset” gaze.

**Fig. 1:**
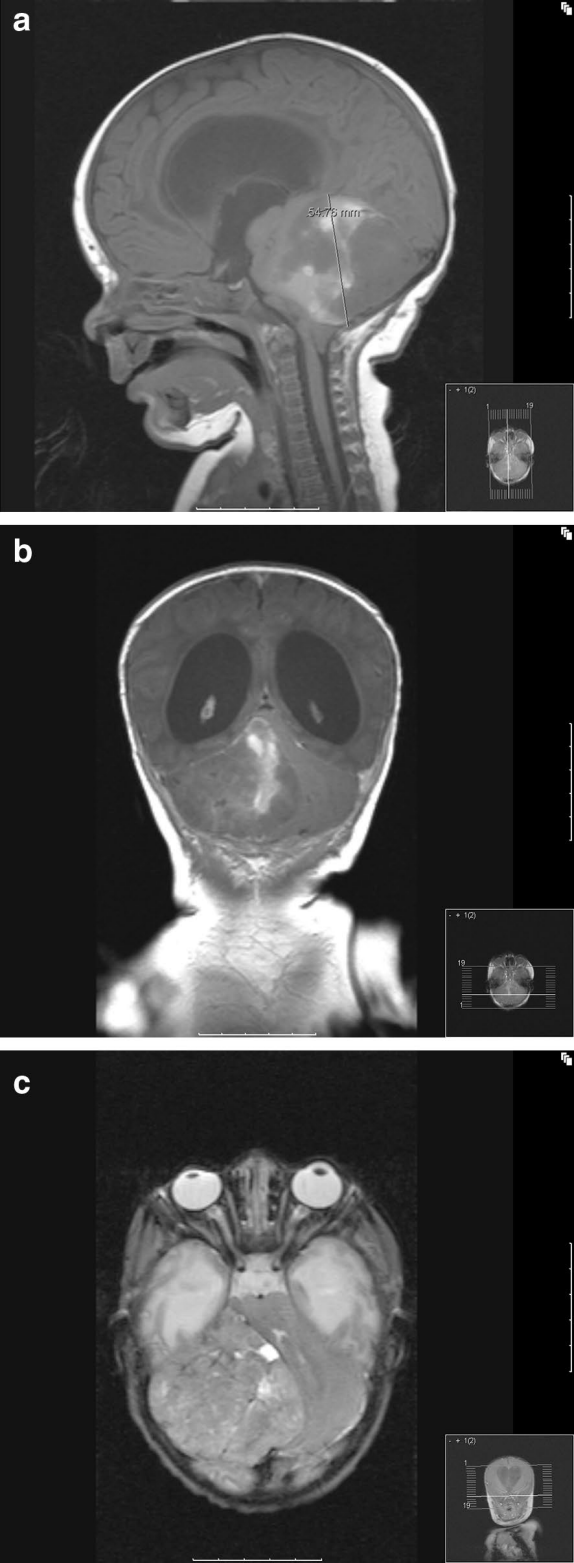
MRI scans disclose a partially contrast enhancing tumor to the right in the posterior fossa with marked displacement of the brain stem to the *left*. **a** sagittal, **b** axial and **c** coronal scans.

*Radiographic findings* An MRI revealed pronounced supratentorial hydrocephalus and a partially contrast enhancing right-sided posterior fossa tumor with downward herniation through the foramen magnum and lateral displacement of the brain stem (Fig. 1).

**Fig. 2:**
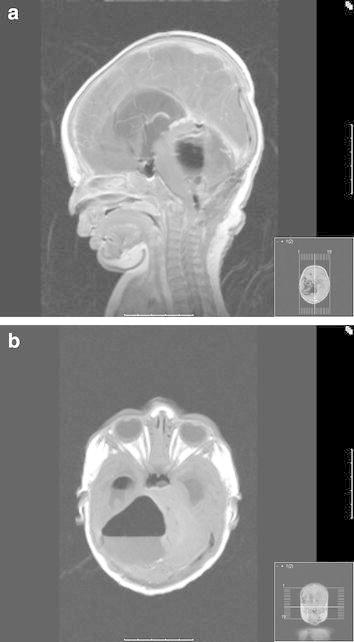
Immediate postoperative MRI scans; **a** sagittal, **b** axial, *white line* demonstrating gross total resection.

*Operation* Intraoperative external drainage of the cerebrospinal fluid (CSF) was established to control the intracranial pressure (ICP). A suboccipital craniotomy was performed in the mid-line. The tumor was microsurgically excised via the fourth ventricle to gross-total resection (GTR) of the tumor. The external drainage was terminated as CSF flow through the Sylvian aqueduct had been observed. Postoperative MRI scans performed in the same anesthetical procedure as the surgery confirmed the GTR (Fig. 2).

**Fig. 3:**
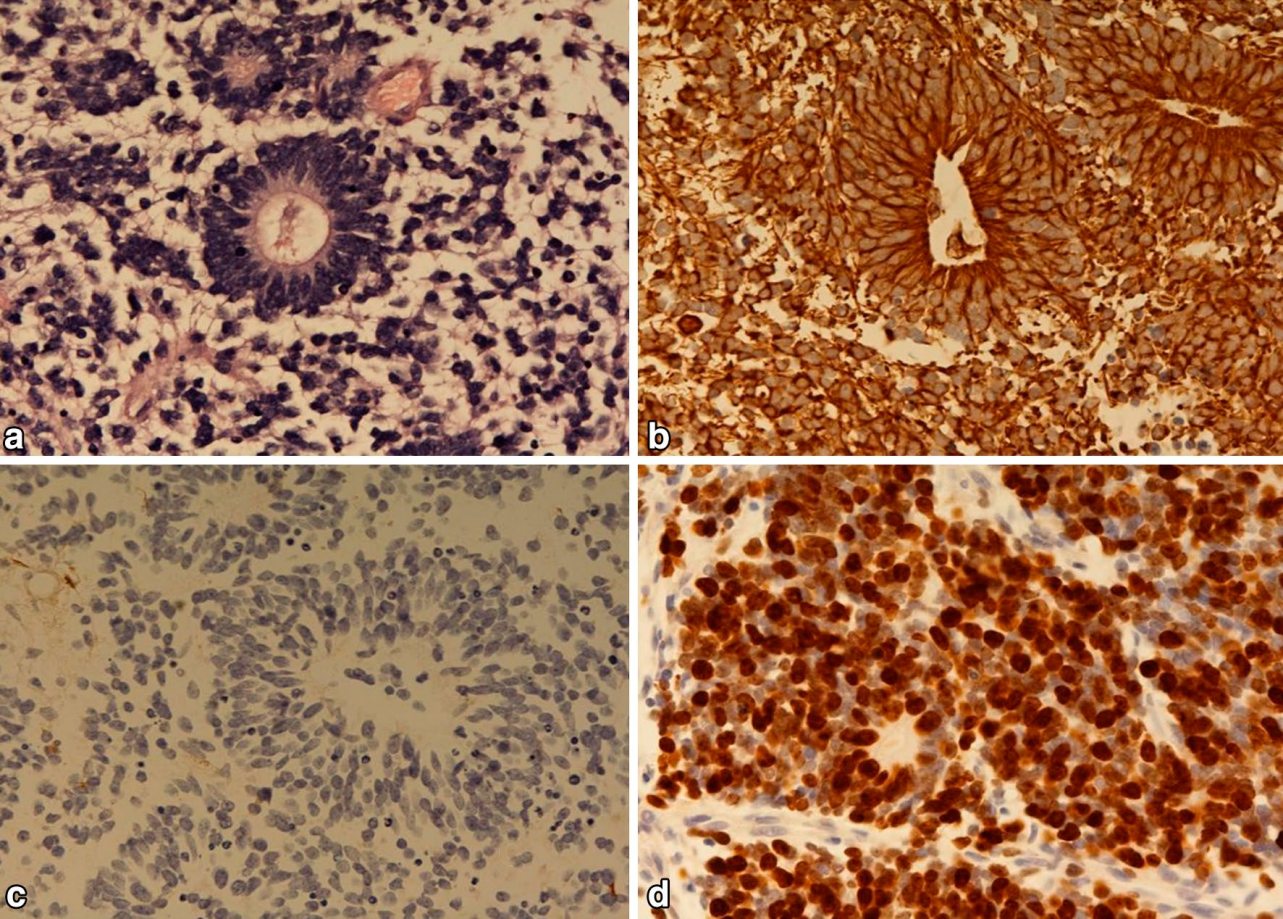
A PNET-like tumor with true rosettes is seen in the HE-stained section (**a**). The true rosettes are positive for vimentin (**b**) and negative for GFAP (**c**). The Ki-67 labelling index is about 90% (**d**).

*The histological examination* revealed a malignant small cell tumor with several true multilayered rosettes (Fig. 3a). The rosettes were postitive for vimentin (Fig. 3b), but negative for GFAP (Fig. 3c) and synaptophysin. The tumor was also focally positive for cytokeratin. The Ki-67 labelling index was about 90% in the most cellular areas (Fig. 3d). The tumor was diagnosed as an ependymoblastoma.

The biopsy has been re-examined by an experienced neuropathologist with the same conclusion.

### Postoperative course and further treatment

Signs of persistent hydrocephalus reappeared after 3 days, and the patient underwent an endoscopic 3rd ventriculocisternostomy. This procedure did not suffice and a ventriculoperitoneal CSF-shunt had to be inserted after 2 weeks. After careful consideration the boy was given chemotherapy according to the MET-HIT 2000-BIS4 protocol during the following 40 weeks. He had several episodes with serious infections, including shunt infection. Following completion of the chemotherapy and infection controls, a new CSF shunt, now ventriculoatrial, was implanted, 14 months after the tumor resection.

**Fig. 4:**
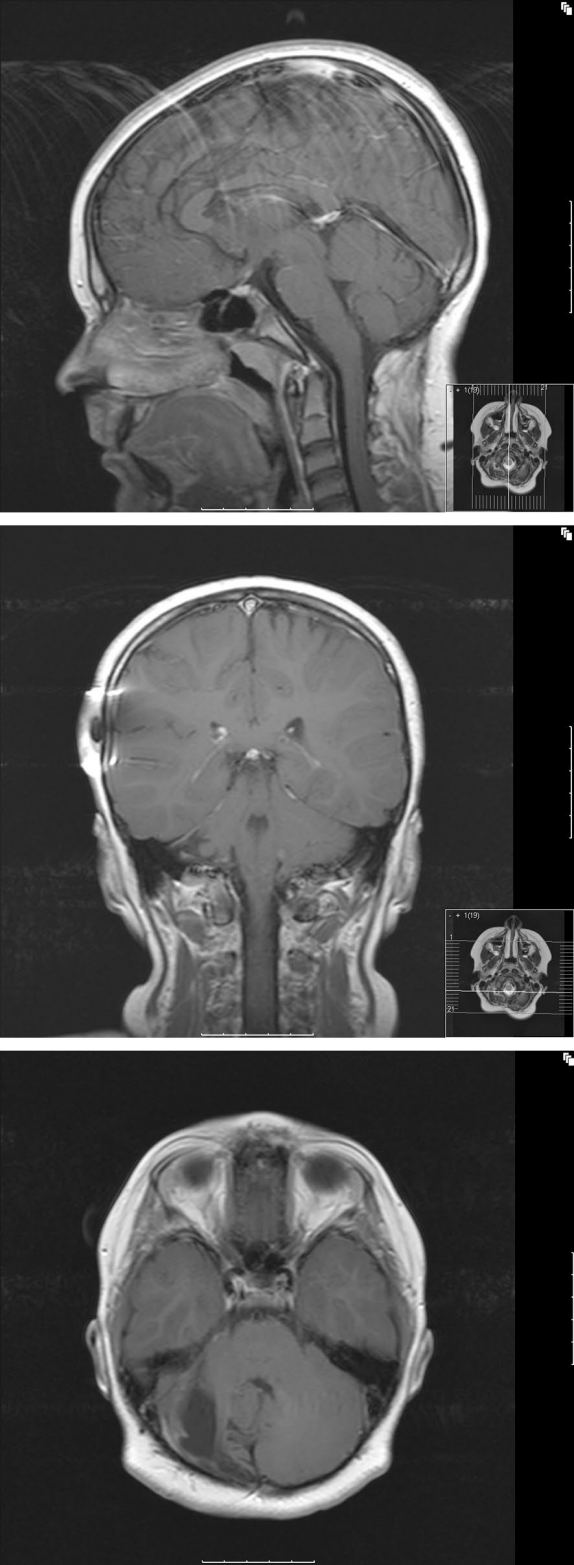
Late follow-up scans after 11 years. Normalisation in the posterior fossa, without signs of residual or recurrent disease.

Serial follow-up MRI scans have demonstrated relative normalization in the posterior fossa (Fig. 4), without any signs of recurrent neoplastic disease, as well as stable ventriculoatrial shunt function.

Today, this boy is 12 years and he follows a normal school program with some assistance (5 h special teaching a week).

He has minor balance problems, but is playing soccer and he is also doing cross-country skiing.

## Discussion

Ependymoblastoma is a rare subentity of PNET with ependymal differentiation and particularly poor prognosis first described by Rubinstein (1970). Most reports are of single cases, but a consecutive series of 11 children aged 1.6–5.6 years was presented by Gerber et al. (2011) They found only three cases (aged 3.4; 3.5 and 5.6 years at diagnosis) with disease free survival, and all these three were among the seven children where GTR had been achieved at primary surgery. Two of these had both postoperative radiotherapy and chemotherapy and were disease free at 12.7 and 9.4 years, the latter had developed a papillary thyroid carcinoma at the latest follow-up. The last child had complete continuous remission for 2.2 years after GTR and high dose chemotherapy (HIT 2000 with autologous stem cell transplantation).

Gerber and colleagues argue for multimodal treatment including radiotherapy to improve survival (Gerber et al. 2011). In our case, the patient was a 2-month-old infant and, in our opinion, too young for radiotherapy.

In their literature review, Ding and coworkers discuss treatment strategies for ependymoblastoma based on the 42 of 72 published cases wherefrom detailed information was available (Ding et al. 2014). They underscore the importance of GTR followed by chemotherapy. Radiation therapy should be given up-front, as this treatment modality given at a later stage does not seem to improve prognosis. They also discuss low age as a negative prognostic factor. This might be caused by biologically more aggressive tumors in infants, or by the fact that infants do not receive radiotherapy.

## Conclusion

Our patient has an uneventful survival for 12 years following GTR and chemotherapy according to the HIT 2000 protocol. To our knowledge, this is the first infant with long-term survival treated for ependymoblastoma.
